# Host prenylation promotes membrane association of the Salmonella effector GtgE during infection

**DOI:** 10.1099/mic.0.001744

**Published:** 2026-07-30

**Authors:** Natalia Cattelan, Hongjiao Yu, Kornelia Przybyszewska, Virtu Solano-Collado, Rosa Colamarino, Massimiliano Baldassarre, Stefania Spanò

**Affiliations:** 1 Institute of Medical Sciences, School of Medicine, Medical Sciences and Nutrition, University of Aberdeen, Foresterhill, Aberdeen AB25 2ZD, UK; 2 Institute of Education in Healthcare and Medical Sciences, School of Medicine, Medical Sciences and Nutrition, Aberdeen, UK

**Keywords:** bacterial effector, GtgE, prenylation, Rab32, *Salmonella*

## Abstract

The Rab32 antimicrobial pathway restricts *Salmonella* Typhi replication in murine macrophages. The broad-host pathogen *Salmonella* Typhimurium has evolved a solution to disrupt this pathway via effector proteins, including GtgE. The presence of a C-terminal CaaX motif in GtgE prompted the hypothesis that the effector was prenylated by the host. Here, we show that the CaaX motif enhances GtgE membrane association and intracellular targeting. Substitution of the C-terminal cysteine (C225S) caused diffused cytosolic distribution and loss of membrane enrichment. Inhibition of host isoprenoid biosynthesis or geranylgeranyl transferase I activity also disrupted GtgE localization. During infection, translocated GtgE was enriched in membrane fractions and accumulated around intracellular *Salmonella* in a pattern consistent with association to the *Salmonella*-containing vacuole, whereas the C225S variant showed reduced membrane association. These findings identify host prenylation as a mechanism that may direct GtgE to intracellular membranes where Rab32 is accessible for cleavage.

## Introduction

*Salmonella enterica* comprises numerous serovars that cause a broad range of diseases. Common gastrointestinal diseases are caused by broad-host range serovars such as *Salmonella* Typhimurium [1], whereas an invasive systemic life-threatening disease known as Typhoid fever is associated with the human-restricted serovar *Salmonella* Typhi [2]. An important trait of *Salmonella* pathogenesis is the intracellular survival of the bacterium in macrophages, dendritic cells and epithelial cells by establishing a replicative niche either inside a vacuole known as the *Salmonella*-containing vacuole (SCV) or in the cytosol of the cells [3]. To successfully do this, the bacterium is equipped with two Type III Secretion Systems (T3SS) encoded in two *Salmonella* pathogenicity islands (SPI), SPI-1 and SPI-2, and a battery of effectors [4].

Host restriction of *S*. Typhi in murine macrophages is partly due to the Rab32 antimicrobial pathway [5]. Rab32, a membrane-associated small GTPase, is recruited to the SCV to deliver itaconate, an antimicrobial compound produced in the mitochondria [5–8]. *S*. Typhimurium counteracts this pathway through two effectors: SopD2, a Rab32 GTPase activating protein (GAP), and GtgE, a cysteine protease that cleaves Rab32 [5, 6]. In contrast, *S*. Typhi lacks both these effectors and is susceptible to Rab32-dependent restriction in mouse macrophages [5, 6].

Rab32 cycles on membranes between GTP- and GDP-bound states and requires a C-terminal geranylgeranylation motif for membrane association [9, 10]. Inactive Rabs can be targeted by GDP dissociation inhibitor (GDI), solubilizing them into the cytosol [9, 10]. Many host and pathogen proteins containing a C-terminal CaaX motif undergo prenylation, a lipid modification that promotes membrane targeting [11–13]. Notably, GtgE contains a C-terminal CTIL sequence consistent with a CaaX prenylation motif. This raises the possibility that the host prenylation machinery may regulate the intracellular localization of GtgE.

Here, we investigated whether GtgE is targeted to membranes through a CaaX-dependent mechanism and determined whether the host prenylation machinery contributed to subcellular localization of GtgE during infection.

## Methods

### Strains and growth conditions, plasmids and primers

All bacterial strains, plasmids and primers used in this work are listed in Tables 1 and 2. *S*. Typhimurium strains were grown on LB (Lennox) agar. When appropriate, antibiotics were used at the following concentrations: streptomycin 50 µg ml^−1^; kanamycin 30 µg ml^−1^ and tetracycline 12.5 µg ml^−1^. Liquid cultures were grown on LB broth or LB broth with 0.3 M NaCl for induction of SPI-1 T3SS genes.

**Table 1.** Strain and plasmids used in this study

Strain, plasmid and primer	Description or sequence	Reference
Strains		
*Salmonella* Typhimurium SL1344	wt genotype, Sm^r^	[25]
*Salmonella* Typhimurium ::*glmS::mCherry*	SL1344 derivative, with genome-inserted *mCherry* coding sequence, Sm^r^	[26]
*S*. Typhimurium *ΔsopD2*	SL1344 derivative, *sopD2* knockout mutant strain, Sm^r^	[27]
*S*. Typhimurium *ΔgtgE*	SL1344 derivative, *gtgE* knockout mutant strain, Sm^r^	[19]
*E. coli* DH5α	*E. coli* laboratory strain	–
Plasmids		
pEYFP-C3	Vector for gene expression in mammalian cells	–
pSB4149	pEYFP-C3-GtgE. GtgE coding sequence was retrieved from SL1344 and cloned into pEYFP-C3 digested EcoRI/SalI	This study
pSB4172	pEYFP-C3-GtgE^C225S^. GtgE^C225S^ coding sequence was retrieved from *S*. Typhimurium *gtgE*^C225S^ and cloned into pEYFP-C3 digested EcoRI/SalI	This study
pSB4829	pSB4004 derivative for expression of *sopD2* 3xFLAG tagged	[6]
pSBA065	pSB4004 derivative for expression of *gtgE* 3x-FLAG tagged under *rpsM* constitutive promoter	This study
pSBA069	pSB4004 derivative for expression of *gtgE^C225S^* 3x-FLAG tagged under *rpsM* constitutive promoter	This study
pSB4004	pWSK^rpsM^-mCherry	[28]

**Table 2.** Primers used in this study

Primer	Sequence	Purpose
Over-GtgE-A	5’ GATCGGATCCATGTTAAGACACATTCAAAATAG	Forward primer to generate pSB4149 and pSB4172 inserts
Over-GtgE-B	5’ GATCTCTAGATCATAAAATGGTACACCAGTC	Reverse primer to generate pSB4149 insert
Over-GtgE-C	5’ GATCTCTAGATCATAAAATGGTAGACCA**C**TC	Reverse primer to generate pSB4172 insert
F-SD-GtgE-EcoRI	5’ TCAGGAATTCGGCGAGTATATTATGT	Forward primer to generate pSBA065 and pSBA069 inserts
F-GtgE-D57	5’ATCCTTGTAATCGATGTCATGATCTTTATAATCACCGT- CATGGTCTTTGTAATCTCTGGATTTGATAACATTTAATGAGC	primer to insert 3xFLAG sequence in D57 position of GtgE
R-GtgE-D57	5’GATTACAAAGACCATGACGGTGATTATAAAGATCATGACA- TCGATTACAAGGATGACGATCTCGGCAATAATTATAGTGCATTAGAG	primer to insert 3xFLAG sequence in D57 position of GtgE
R-GtgE-KpnI	5’AGCTGGTACCTCATAAAATGGTAGAC	Reverse primer to generate pSBA065 insert
R-GtgE-C225S-KpnI	AGCTGGTACCTCATAAAATGGTACAC	Reverse primer to generate pSBA069 insert

### Cell lines

HeLa cells (ATCC^®^ CCL-2™) were grown in Dulbecco’s modified Eagle’s medium (DMEM high glucose with GlutaMAX, Gibco) supplemented with 10% FBS (Gibco) and cultured in 10 cm tissue culture (Fisher) plastic plates at 37 °C, 5% CO_2_.

### Plasmid constructs generation and DNA manipulation

#### Overexpression constructs

For overexpression assays in HeLa cells, *gtgE* and *gtgE^C225S^* were amplified with primers pair Over-GtgE-A and Over-GtgE-B, and Over-GtgE-A and Over-GtgE-C (including the G-to-C mutation that generated the C225S mutation), respectively, from *S*. Typhimurium wt. These PCR fragments were then digested with BamHI and XbaI and cloned into BamHI/XbaI digested pEYFP-C3 plasmid, to generate constructs pSB4149 (YFP-GtgE) and pSB4172 (YFP-GtgE^C225S^). Constructs were transformed into electrocompetent *E. coli* DH5α cells, selected on kanamycin and confirmed by sequencing.

#### Insertion of 3xFLAG tag into GtgE and GtgE^C225S^

A 3xFLAG tag was introduced at Asp^57^ position of GtgE by overlapping PCR with primers F-SD-GtgE-EcoRI, F-GtgE-D57, R-GtgE-D57, R-GtgE-KpnI (for *gtgE* wt sequence) or R-GtgE-C225S-KpnI (for *gtgE^C225S^* sequence). Amplicons were digested with EcoRI/KpnI and cloned into EcoRI/KpnI digested pSB4004, to generate pSB065 (for *gtgE*-3xFLAG) and pSB069 (for *gtgE^C225S^*-3xFLAG). Constructs were transformed into electrocompetent *E. coli* DH5α cells and selected on kanamycin. Correct inserts were confirmed by sequencing and then electroporated into *S*. Typhimurium strains.

### Intracellular localization of GtgE

HeLa cells were seeded onto #1 glass coverslips (Thermo Scientific) in 24-well plates at a density of 2×10^4^ cells and grown overnight. Cells were transfected using polyethylenimine (PEI; 60 µg ml^−1^, Sigma-Aldrich) with 1 µg of DNA (pSB4149 or pSB4172). DNA and PEI were incubated separately in serum-free DMEM for 10 min, then mixed in a 1 : 1 ratio, incubated for additional 20 min at room temperature and then added to the cells. After 24 h, cells were fixed with 4% Paraformaldehyde (PFA).

Where indicated, transfected cells were treated with the following inhibitors: mevastatin (lipid biosynthesis inhibitor, 10 µM, Merck), L744,832 (farnesyl transferase inhibitor, 5 µM Merck) and GGTI-298 (geranylgeranyl transferase I inhibitor, 1 µM, Merck).

For infection assays, transfected cells were infected with chromosomally mCherry-expressing *S*. Typhimurium at an m.o.i. of 20 following the gentamicin protection assay protocol as previously described [6]. At 2.5 hours post-infection (hpi), cells were washed, fixed and mounted on coverslips using ProLong Diamond antifade reagent (Thermo Fisher). Samples were imaged using a Zeiss LSM880 confocal microscope, acquiring z-stacks and generating maximum-intensity projections. Experiments were performed twice with duplicate samples.

### Subcellular fractionation

Subcellular fractionation was performed by multi-step centrifugation according to Gomes *et al*. with minor modifications [14]. HeLa cells were transfected to express either YFP-GtgE wt or YFP-GtgE^C225S^ with plasmids pSB4149 and pSB4172, respectively, as described before. Cells were seeded at a density of 5×10^5^ cells in 10 cm culture dishes and grown overnight. In addition, fractionation was performed on infected HeLa cells with *S*. Typhimurium harbouring either pSB065 or pSB069 at an m.o.i. of 20 at 4 hpi.

Cells were washed twice with warm PBS, harvested by trypsinization and suspended in homogenization buffer (250 mM sucrose, Sigma-Aldrich; 10 mM HEPES pH 7.4, Sigma-Aldrich; 1 mM EDTA, Sigma-Aldrich, 2 mM PMSF, Roche). Homogenization was performed by a single freeze/thaw cycle, followed by repeated passage of the cellular material through a 25-gauge needle. An aliquot was retained as total lysate. Debris was removed by low-speed centrifugation at 500 ***g*** for 10 min at 4 °C. Subsequently, the supernatant was centrifuged at 100,000 ***g*** for 1 h at 4 °C; however, infected HeLa were subjected to centrifugation at 10,000 ***g*** for 10 min at 4 °C prior to the ultracentrifugation step. Each of the fractionation steps including pellets after centrifugations at 500 ***g*** (PN sample), 10,000 ***g*** (MID sample) and 100,000 ***g*** (membrane fraction) were suspended in 100 µl of 2X loading buffer [4% SDS, 10% β-mercaptoethanol, Sigma-Aldrich, 20% (v/v) glycerol, Sigma-Aldrich, 125 mM Tris, Sigma-Aldrich and 0.004% bromophenol blue, Sigma-Aldrich], whilst the ultracentrifugation supernatant (cytosol fraction) was precipitated with TCA prior to resuspension. Equal volumes were analysed by SDS-PAGE and immunoblotting. Experiments were repeated at least three times.

### Translocation

Translocation of T3SS effectors was assessed as previously described [7]. Briefly, HeLa cells were seeded at 5×10^5^ in a six-well plate and grown overnight. Cells were infected with *S*. Typhimurium wt, *S*. Typhimurium *ΔgtgE* pSB065 (encoding GtgE-3xFLAG), *S*. Typhimurium *ΔgtgE* pSB069 (encoding GtgE^C225S^-3xFLAG) and *S*. Typhimurium *ΔsopD2* pSB4829 (encoding SopD2-3xFLAG, as a positive control) at an m.o.i. 20 for 5 h, following the gentamicin protection assay. Proteinase K (30 µg ml^−1^) suspended in Hank’s balanced salt solution (HBSS) was added 15 min prior to the infection end point to detach the cells by incubation at 37 °C and was inhibited by addition of 2 mM PMSF solution in HBSS. Cells were harvested and lysed in ice-cold 0.2% Triton X-100 (Sigma-Aldrich) in PBS and 2 mM PMSF and centrifuged at 20,000 ***g*** for 5 min at 4 °C. Supernatant was collected and filtered through a Millipore Millex LG Syringe Filter with hydrophilic low chemical resistance polytetrafluoroethylene (LCR PTFE membrane) (0.2 µm) by centrifugation at 20,000 ***g***, 30 s at 4 °C. Pellet and filtered fractions were analysed by SDS-PAGE and immunoblotting.

### *In vitro* secretion assay

Bacterial strains expressing GtgE-3 ×FLAG, GtgEC225S-3×FLAG or empty vector were grown overnight, diluted 1 : 20 in LB containing 0.3 M NaCl and cultured to an OD_600_ of 0.9. Culture supernatants (2 ml) were TCA-precipitated and resuspended in sample buffer. Corresponding bacterial pellets were lysed in sample buffer. Samples were analysed by SDS-PAGE and immunoblotting using anti-FLAG antibodies. Bacterial counts were determined retrospectively by plating serial dilutions.

### Western blotting

Protein samples were separated by 12.5% SDS-PAGE, transferred to PVDF membranes (Immobilon; Millipore) with a semi-dry system (Bio-Rad) and then subjected to immunochemical detection. Primary antibodies were prepared in 2.5% skimmed milk and Tris-buffered saline (TBS)-0.05% Tween (TBST), at the following dilutions: rabbit anti-GFP dilution (1 : 3,000, Abcam ab6556); rabbit anti-α-actin (1 : 1,000, Cell Signalling 4967), mouse anti-mouse Rab32 (1 : 1,000, Santa Cruz sc-390178); mouse monoclonal anti-FLAG M2 (1 : 5,000, Sigma F3165); anti-CD29 (1 : 1,000, BD Biosciences Clone 18). Blocked membranes were incubated overnight at 4 °C with the respective primary antibody. Next, the corresponding secondary antibodies prepared at the following dilutions were added and incubated for 1 h at room temperature: donkey anti-rabbit-800 (1 : 15,000, LI-COR 926-32213), donkey anti-mouse-800 (1 : 15,000, LI-COR 926-32212) and goat anti-mouse HRP (1 : 10,000, Sigma A4416). Blots were developed using either ECL-detection method (Thermo Fisher) or the Odyssey infrared imaging system (LI-COR Biosciences).

## Results

### GtgE membrane localization is promoted by a C-terminal CaaX motif

**Fig. 1.**
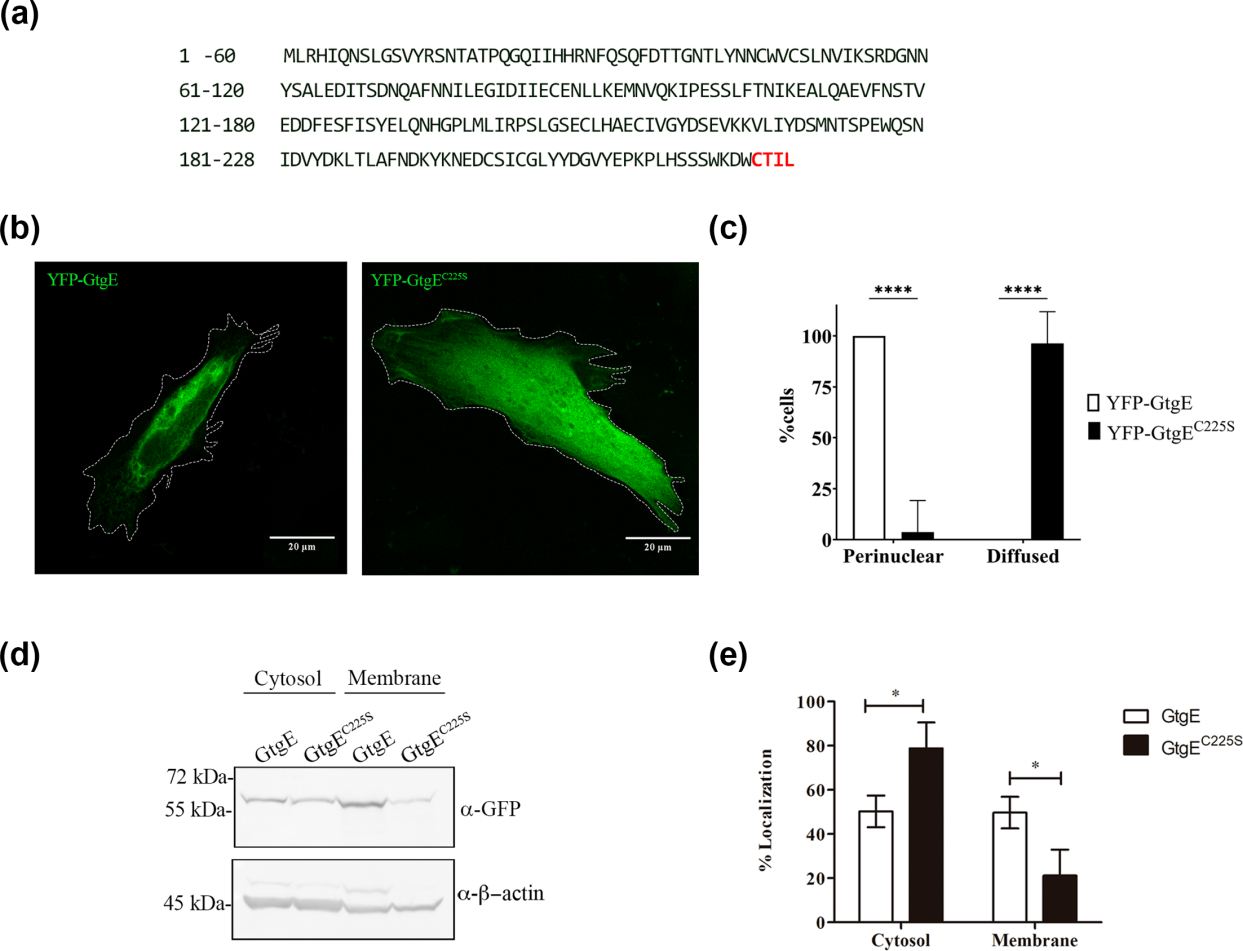
The C-terminal CaaX cysteine enhances membrane association of GtgE. (**a**) Sequence of GtgE showing the predicted CaaX motif (CTIL) in red at the C-terminus. (**b**) Substitution of GtgE Cys225 with Serine (C225S) affects GtgE intracellular distribution. HeLa cells were transfected to express YFP-GtgE or YFP-GtgE^C225S^ (green) and fixed at 24 hr after transfection. Representative maximum-intensity projections of confocal z-stacks show a perinuclear-associated localization of YFP-GtgE (left panel) and a diffused cytosolic and nuclear distribution of YFP-GtgE^C225S^ (right panel). Scale bar: 20 µm. (**c**) GtgE distribution patterns in HeLa transfected cells to express YFP-GtgE or YFP-GtgE^C225S^. Data are shown as mean percentages±sd from three independent experiments. Statistical significance was assessed by Student’s t-test; ****P<0.0001. (**d**) Subcellular fractionation of transfected HeLa cells. Enriched cytosolic and membrane fractions were prepared from HeLa cells expressing YFP-GtgE or YFP-GtgE^C225S^. GtgE was detected with an anti-GFP antibody. β-actin was used as internal loading control. (**e**) Western blot densitometry semi-quantification. Data are shown as mean±sem from three independent experiments, with percentage distribution across fractions indicated. Statistical significance was assessed by Student’s t-test; *P<0.05.

*Salmonella* uses different host systems of post-translational modification to modify effectors for its own benefit and survival [5]. Sequence analysis revealed a C-terminal CTIL motif in GtgE, consistent with a conserved CaaX prenylation sequence (Fig. 1a). This motif can be recognized and targeted by host enzymes to introduce a hydrophobic isoprenoid onto the cysteine [12]. Once prenylated, proteins can be redirected to membranes within the cell [12]. We hypothesized that if GtgE is prenylated by the host, it would localize to Rab32-positive membrane compartments, where Rab32 undergoes inactivation by SopD2 and is accessible outside the protective Rab32–GDI cytosolic complex [10]. To test whether the CaaX motif influences GtgE intracellular localization, we generated a variant that cannot be prenylated by substituting Cys^225^ with a Serine (GtgE^C225S^). This amino acid substitution has been previously used in prenylation studies [6].

HeLa cells expressing YFP-GtgE displayed a predominantly perinuclear distribution (Fig. 1b, c). In contrast, YFP-GtgE^C225S^ was diffusely distributed throughout the cytoplasm and nucleus (Fig. 1b, c). This suggests that the C-terminal cysteine contributes to localization of GtgE.

To confirm this finding, we performed subcellular fractionation of transfected cells (Fig. 1d, e). Wild-type (wt) GtgE was enriched in membrane fractions, whereas the C225S variant showed increased localization in the cytosolic fraction and reduced detection in enriched membrane fractions (Fig. 1d, e). These results suggest that the CaaX cysteine enhances membrane association of GtgE.

### Host isoprenoid biosynthesis and GGTase-I activity contribute to GtgE intracellular localization

**Fig. 2.**
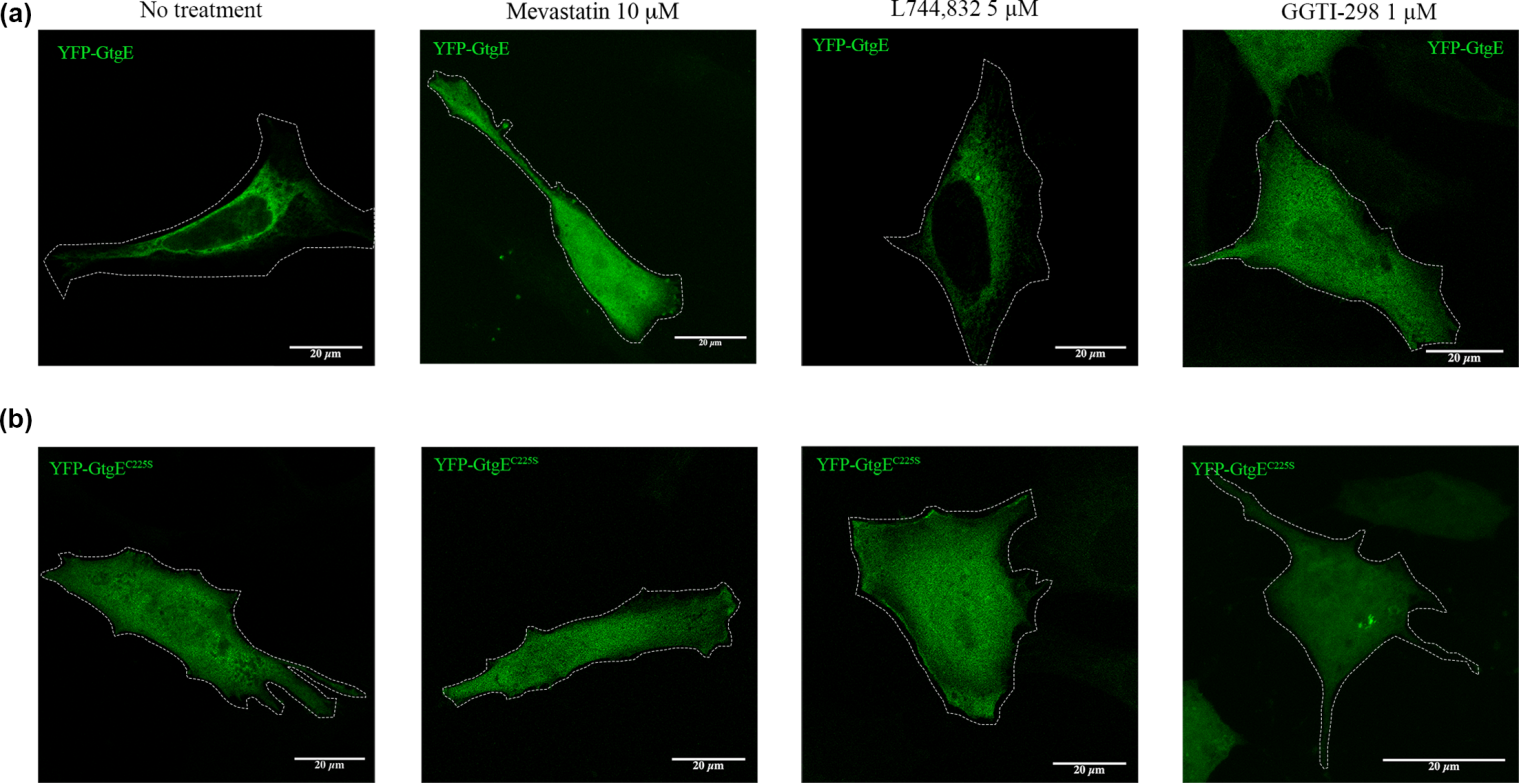
Inhibition of host isoprenoid biosynthesis and geranylgeranyl transferase I activity alters GtgE intracellular localization. HeLa cells were transfected to express YFP-GtgE (**a**) or YFP-GtgE^C225S^ (**b**) (green) and treated with the indicated inhibitor: mevastatin (10 µM), L-744,832 (5 µM) or GGTI-298 (1 µM). Cells were fixed 24 h post-transfection. Representative maximum-intensity projections of confocal z-stacks are shown. Inhibition of isoprenoid biosynthesis or geranylgeranyl transferase I activity disrupted membrane localization of YFP-GtgE, resulting in diffused cytosolic distribution, whereas no effect was observed on YFP-GtgE^C225S^. Scale bar: 20 µm.

To further investigate whether the host prenylation machinery contributes to GtgE localization, we inhibited isoprenoid biosynthesis by mevastatin treatment of transfected HeLa cells. Under these conditions, YFP-GtgE lost its perinuclear distribution pattern and became diffusely cytosolic in 100% of visualized cells, resembling the C225S mutant (Fig. 2a). No effect was observed on YFP-GtgE^C225S^ localization following mevastatin treatment (Fig. 2b).

We next used selective inhibitors of farnesyl transferase (L-744,832) and geranylgeranyl transferase I (GGTI-298). Inhibition of GGTase-I disrupted the perinuclear localization pattern of wt GtgE in 100% of visualized cells, whereas inhibition of farnesyl transferase had no detectable effect in any of the visualized cells (Fig. 2a). Neither inhibitor altered the localization pattern of YFP-GtgE^C225S^ (Fig. 2b). These findings suggest that host geranylgeranyl transferase activity contributes to the intracellular localization of GtgE.

### CaaX-dependent lipidation enables GtgE recruitment to the SCV

**Fig. 3.**
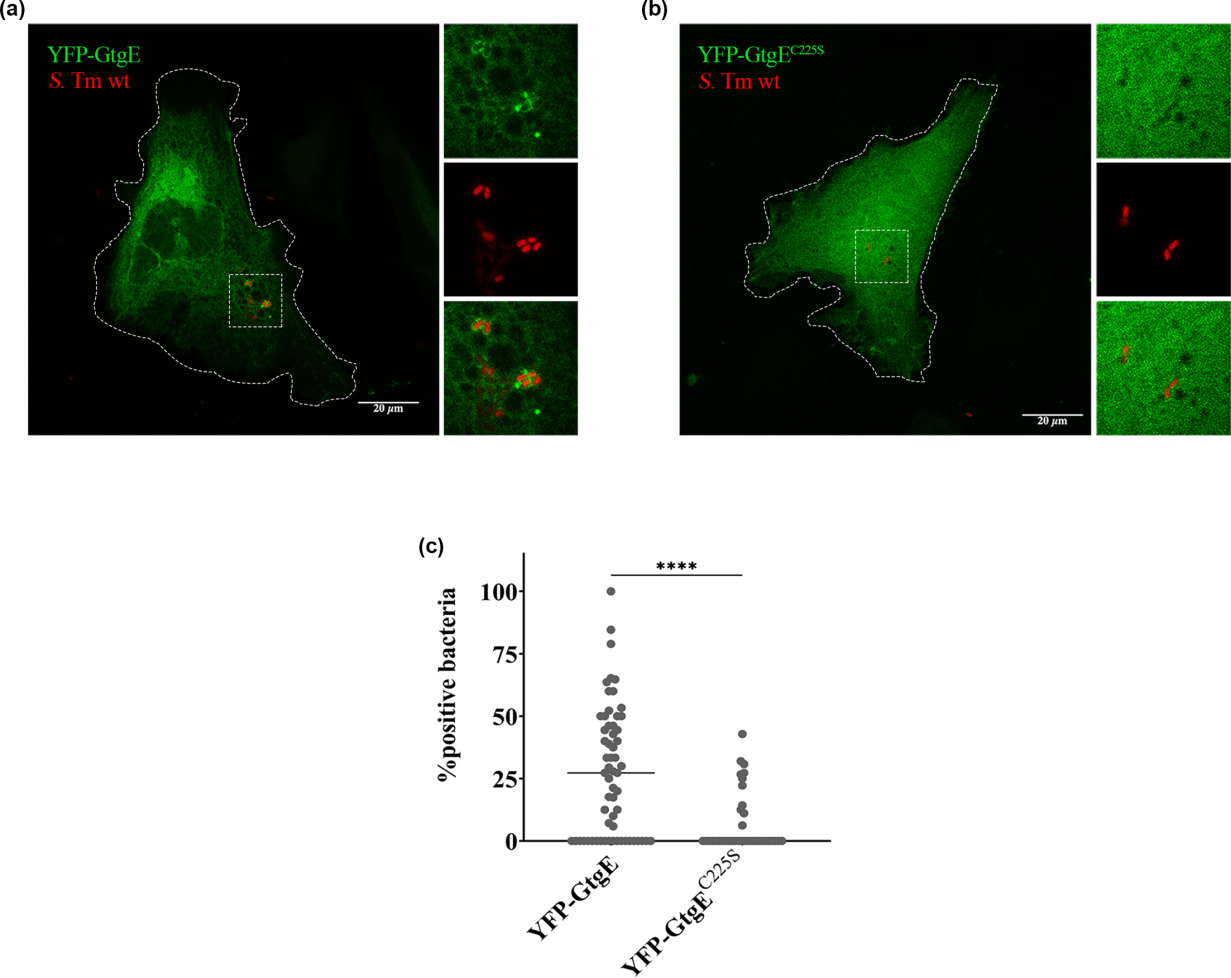
The CaaX motif promotes association of GtgE with intracellular *Salmonella.* HeLa cells expressing YFP-GtgE (**a**) or YFP-GtgE^C225S^ (**b**) (green) were infected with mCherry-expressing *S*. Typhimurium (red) for 2.5 h. Representative maximum-intensity projections of confocal z-stacks are shown. Scale bar: 20 µm. (**c**) Quantification of bacteria that recruit YFP-GtgE and YFP-GtgE^C225S^. Each data point represents the percentage of positive bacteria within one cell. Statistical significance from three independent experiments was assessed by Student’s t-test; ****P<0.0001.

We next investigated the role of GtgE prenylation on subcellular localization of the effector protein during infection. HeLa cells expressing YFP-GtgE or YFP-GtgE^C225S^ were infected with mCherry-labelled *S*. Typhimurium (Fig. 3a, b). Wt GtgE showed a perinuclear distribution pattern and accumulation at the proximity of intracellular bacteria, indicating a possible recruitment to the SCV membrane (Fig. 3a). In contrast, the C225S variant remained diffusely distributed (Fig. 3b). We quantified the proportion of intracellular bacteria that recruits YFP signal, observing a significant increase of YFP-positive bacteria in cells expressing YFP-GtgE (27%) compared to YFP-GtgE^C225S^ (7%) (Fig. 3c).

**Fig. 4.**
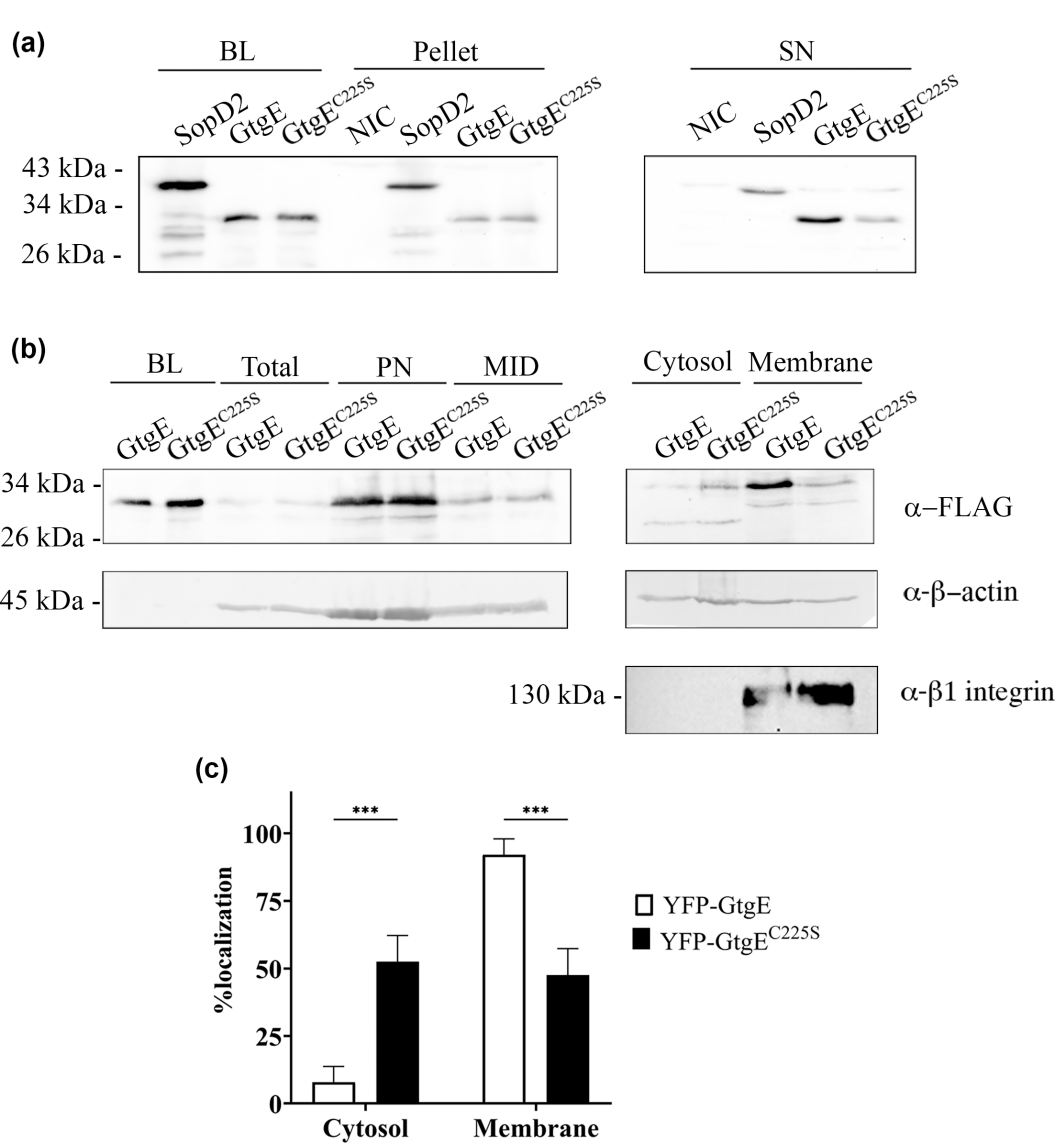
Translocation of 3xFLAG-tagged GtgE variants and subcellular distribution of translocated GtgE during infection. (**a**) HeLa cells were infected with an m.o.i. of 20 with *S*. Typhimurium *ΔsopD2* pSB4829 (SopD2-3xFLAG as positive control), *S*. Typhimurium *ΔgtgE* pSB065 (GtgE-3xFLAG) or S. Typhimurium *ΔgtgE* pSB069 (GtgE^C225S^-3xFLAG). After 5 hours post-infection (hpi), cells were lysed and centrifuged at 10,000 ***g*** for 10 min. The supernatant (SN) was filtered through a 0.2 µm filter to eliminate any remaining bacteria, and the SN and the cell pellet (Pellet) were used for Western blot analysis, with respective bacterial lysate (BL) from the inoculum used for infection serving as positive control. NIC, non-infected control. Blots were developed with an anti-FLAG antibody. (**b**) HeLa cells were infected at an m.o.i. of 20 with *S*. Typhimurium *ΔgtgE* pSB065 (GtgE-3xFLAG) or *S*. Typhimurium *ΔgtgE* pSB069 (GtgE^C225S^-3xFLAG). After 4 hpi, cells were collected (Total sample), lysed and subjected to differential centrifugation; each pellet sample was used for Western blot analysis. Postnuclear pellet (PN): represents 500 ***g*** pellet; MID pellet (MID): 10,000 ***g*** pellet; membrane pellet (Membrane): 100,000 ***g*** pellet; cytosol fraction was obtained by TCA precipitation of the resultant SN after 100,000 ***g*** centrifugation (Cytosol). Blots were probed with an anti-FLAG antibody. β-actin and β1 integrin were used as controls. BL represents lysates from the inoculum used for infection. Three independent experiments were performed. (**c**) Western blot densitometry semi-quantification. Data are shown as mean±sd from three independent experiments, with percentage distribution across cytosolic and membrane fractions indicated. Statistical significance was assessed by Student’s t-test; ***P<0.001.

To determine whether GtgE translocated by *S*. Typhimurium was membrane-associated during infection, we introduced a 3xFLAG epitope at Asp^57^ within a loop region of both GtgE and GtgE^C225S^. This insertion site was selected to avoid interference with the C-terminal CaaX motif. The tagged variants were introduced into a *S*. Typhimurium Δ*gtgE* background to ensure that only the tagged protein was delivered during infection. Translocation assays confirmed that both constructs were secreted into the cell cytosol (Fig. 4a). However, GtgE^C225S^ was detected in lower quantities in infected cell lysates, suggesting reduced protein stability and/or translocation efficiency (Fig. 4b).

HeLa cells were subsequently infected with *S*. Typhimurium Δ*gtgE* expressing GtgE-3xFLAG or GtgE^C225S^−3xFLAG, and the cytosolic and membrane-enriched fractions of infected cell lysates were separated by a sequence of increasing speed centrifugation steps.

The bacterial lysate (BL), total cell lysate (Total), postnuclear pellet (PN, representing 500 ***g*** pellet), MID pellet (MID, representing 10,000 ***g*** pellet), membrane pellet (Membrane, representing 100,000 ***g*** pellet) and cytosol fraction (Cytosol, obtained by TCA precipitation of the resultant supernatant after 100,000 ***g*** centrifugation) were analysed by Western blot. Semiquantification by densitometry showed that GtgE-3xFLAG wt was predominantly detected in membrane fractions (92%), while significantly lower levels of GtgE^C225S^ (46%) were detected in the membrane fraction (Fig. 4a, b). We conclude that the C-terminal CaaX motif contributes to membrane enrichment of translocated GtgE during infection.

## Discussion

Manipulating host activities through translocated effectors is crucial for intracellular survival in bacterial pathogens [15]. To achieve specificity within the host cell, several bacterial effectors exploit the host post-translational modification machinery to control their intracellular localization [16, 17]. While modifications such as ubiquitination, phosphorylation or lipidation have been extensively reported [16, 17], the role of host-mediated prenylation in effector targeting remains understudied. By means of protein prenylation, an isoprenoid (farnesyl or geranylgeranyl) is irreversibly added to a Cys residue in a C-terminal CaaX motif [11]. Consequently, a hydrophobic domain is added to the protein, resulting in its localization to an intracellular membrane compartment (reviewed in [13]). Previous bioinformatic analysis performed by Al-Quadan *et al*. showed a list of potential prenylation targets from different bacterial pathogens with a C-terminal CXXX motif [18]. One such protein is GtgE, which contains a CTIL sequence at its C-terminus. GtgE is a *Salmonella* T3SS effector involved in the inactivation of the Rab32 antimicrobial pathway in the host [19]. This feature raised the possibility that the host prenylation machinery could influence the intracellular localization of GtgE during infection.

In this work, we examined the contribution of the C-terminal cysteine within the CTIL motif to GtgE localization. Substitution of Cys^225^ with Serine (C225S) resulted in a redistribution of GtgE from a perinuclear pattern to a diffused cytosolic and nuclear localization in transfected HeLa cells. Prenylation of bacterial effectors has so far been reported in *Legionella pneumophila* (in AnkB and six AA100 Pel proteins) [20, 21] and in *S*. Typhimurium (in SifA) [22]. The same Cys to Ser modification has been previously used to study these effectors [20–22]. We then confirmed the differences in localization by Western blot and subcellular fractionation. Consistent with a role for host prenylation machinery, inhibition of isoprenoid biosynthesis or geranylgeranyl transferase I activity resulted in redistribution of GtgE from perinuclear to diffused. Importantly, this effect was not observed for the C225S variant, supporting the specificity of this mechanism. GtgE was also found in the vicinity of intracellular *Salmonella*. Although direct recruitment to the SCV membrane was not formally demonstrated in this study, this localization pattern is consistent with association to the SCV. Future work including the use of established SCV markers such as LAMP-1, will be required to confirm this. During infection, translocated GtgE was enriched in membrane fractions. While we attempted direct biochemical detection by MS/proteomics of prenylated GtgE in infected cells (data not shown), this proved to be technically challenging due to low effector abundance and the hydrophobic, processed nature of CaaX modifications [23]. Overall, the convergent genetic and pharmacological evidence supports a prenylation-dependent targeting mechanism. We conclude that the C-terminal CaaX motif contributes to membrane enrichment of translocated GtgE during infection.

**Fig. 5.**
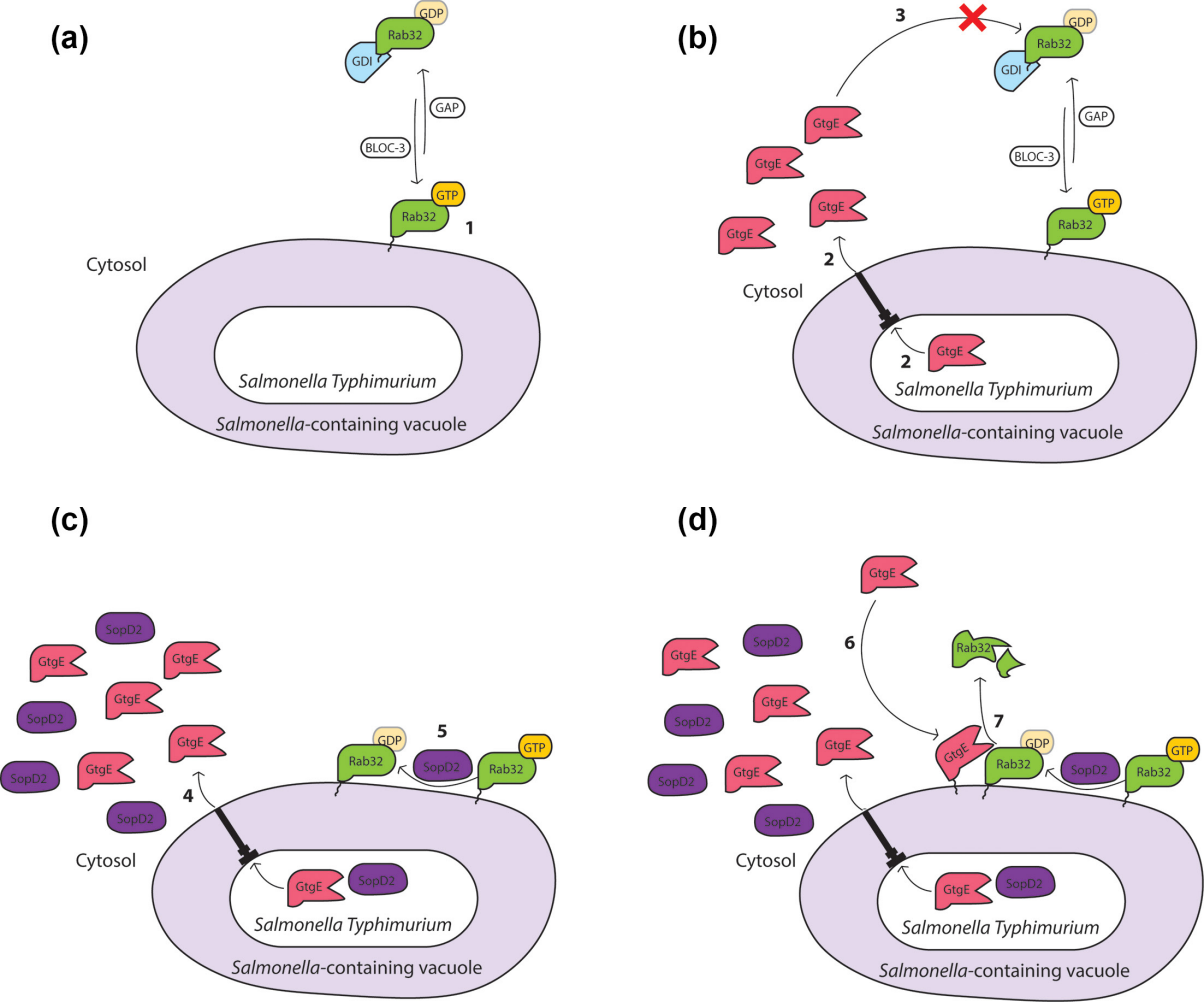
Step-by-step model for CaaX-dependent membrane targeting of GtgE during *Salmonella* Typhimurium infection. (**a**) In uninfected cells, Rab32 cycles between its GDP-bound cytosolic form (complexed with GDP dissociation inhibitor-GDI) and its GTP-bound membrane-associated form, regulated by host factors (e.g. BLOC3 and GTPase activating protein-GAP). Membrane-associated Rab32-GTP (step 1) is the active form recruited to the SCV during infection. (**b**) Following translocation of GtgE by *S*. Typhimurium into the host cytosol (step 2), cytosolic Rab32-GDP bound to GDI is not efficiently targeted by GtgE, as the GDI interaction occludes the cleavage site (step 3, blocked by red cross).(**c**) In addition to GtgE, SopD2 is translocated into the host cell cytosol through the SPI-2 T3SS (step 4). On the SCV membrane, SopD2 acts as a GAP to inactivate membrane-associated Rab32-GTP, converting it to Rab32-GDP (step 5), generating membrane-associated Rab32-GDP accessible to GtgE. (**d**) Host-mediated prenylation of the C-terminal CaaX motif enhances GtgE localization to membranes (step 6), where it encounters membrane-associated Rab32-GDP not shielded by GDI. This increases productive cleavage of Rab32 by GtgE (step 7), disrupting the antimicrobial pathway.

Based on these localization data, we propose a model in which host-mediated prenylation targets GtgE to intracellular membranes, including the SCV (Fig. 5). Rab32, a central component of the antimicrobial pathway targeted by GtgE [6], is a membrane-associated Rab GTPase that cycles between GTP- and GDP-bound states on membranes, whereas cytosolic Rab32-GDP is complexed with the GDI. GtgE cannot target cytosolic Rab32, since its interaction with GDI hinders the cleavage site [24]. Consistent with reports that GtgE preferentially cleaves inactive Rab GTPases, membrane targeting of GtgE may increase productive encounters with Rab32-GDP present on membranes during nucleotide cycling and/or following SopD2-mediated GAP activity. However, due to differences in delivery and abundance of the C225S GtgE variant during infection, this study did not directly assess the functional consequences of membrane targeting on Rab32 cleavage or downstream antimicrobial outcomes.

The use of host machinery to spatially restrict effector activity represents an efficient strategy for bacterial pathogens, allowing low-abundance effectors to act selectively at relevant subcellular sites. Similar mechanisms have been described for the prenylation of effector proteins of intracellular pathogens, including *L. pneumophila* [20, 21] and *Salmonella* Typhimurium [22], suggesting that host-mediated lipidation may represent a conserved strategy for effector targeting.

In summary, our work demonstrates that the C-terminal CaaX motif of GtgE promotes membrane enrichment of the effector, including to the vicinity of intracellular bacteria and identifies host prenylation machinery as a key determinant of its intracellular localization. Our findings provide a framework for understanding how spatial regulation of effector proteins contributes to host–pathogen interactions and highlight the importance of host-mediated lipid modifications in controlling the positioning of effectors within infected cells.

## Abbreviations

BL: bacterial lysate
DMEM: Dulbecco’s modified Eagle’s medium
GAP: GTPase activating protein
GDI: GDP dissociation inhibitor
HBSS: Hank’s balanced salt solution
hpi: hours post-infection
PEI: polyethylenimine
PFA: Parafornaldehyde
PN: postnuclear pellet
SCV: *Salmonella*-containing vacuole
SN: supernatant
SPI: *Salmonella* pathogenicity island
T3SS: Type III Secretion Systems
